# Supplementary material for: The observation of π-shifts in the Little-Parks effect in 4Hb-TaS2
Source: Nat Commun. 2024 May 30;15:4623. doi: 10.1038/s41467-024-48260-x (PMC11139670; doi:10.1038/s41467-024-48260-x)
Supplement: Supplementary file 1 — Supplementary Information [file 41467_2024_48260_MOESM1_ESM.pdf]

# Supplementary Materials for
# The observation of $\pi$-shifts in the Little-Parks effect in 4Hb-$TaS_2$

Avior Almoalem,[1] Irena Feldman,[1] Ilay Mangel,[1] Michael Shlafman,[2] Yuval E. Yaish,[2] Mark H. Fischer,[3] Michael Moshe,[4] Jonathan Ruhman,[5] and Amit Kanigel[1,*]

[1]*Physics Department, Technion-Israel Institute of Technology, Haifa 32000, Israel.*
[2]*Andrew and Erna Viterbi Faculty of Electrical and Computer Engineering, Technion, Haifa 32000, Israel.*
[3]*Department of Physics, University of Zurich, Winterthurerstrasse 190, 8057 Zurich, Switzerland*
[4]*Racah Institute of Physics, The Hebrew University of Jerusalem, Jerusalem 91904, Israel.*
[5]*Department of Physics, Bar-Ilan University, 52900, Ramat Gan, Israel.*

## CONTENTS

Ring fabrication 2
Electron Backscatter diffraction map of a ring 2
Current dependence 3
The amplitude of the Little-Parks oscillations 3
Zero-field calibration of the superconducting magnet 4

Additional data 5
Extended data: observation of the Little-Parks oscillations in eight different devices 6
Thermal cycles 7
In-plane magnetic field 7

Theory 9
Ginzburg-Landau Theory of the two-component order parameter 9
The Little-Parks effect in uniform strain 11
The Little-Parks effect in a chiral state 11
The chiral-nematic mixed state in the presence of strain and magnetic field 12
Possible scenario for half-vortices of the planar strain field: The strain induced by a dislocation 13
Topological classification of the strain field around the ring and its relevance to the Little-Parks experiment 14

References 16

## Ring fabrication

The fabrication steps are shown in Fig.S1. We exfoliate 4Hb-$TaS_2$ flakes on a $SiO_2$/Si substrate using the standard dry transfer technique (Fig.S1a). We cover the flakes with a ∼500 nm protective layer of $SiO_2$ *in-situ* (Fig. S1b) before using a FEI Helios NanoLab DualBeam G3 UC focused ion beam (FIB) to carve the desired ring from the exfoliated flake (Fig. S1c). For carving we use a 30 kV, 40 pA current. All the rings have the same shape and only the sizes vary. SEM images of several rings are shown in Fig. S2.

## Electron Backscatter diffraction map of a ring

As explained in the main text, the $\pi$-shift may appear in polycrystalline samples if three or more grain boundaries are formed with the right angle and the order parameter is nodal and non-s-wave [1]. Thus, it is paramount to understand the crystallographic structure of our samples. While they are fabricated from single crystals it is important to understand the influence of the fabrication process, and whether it introduces such grain boundaries.

Unfortunately, our devices are coated with $SiO_2$, which does not allow a direct measurement of crystallographic structure of the partnered 4Hb-$TaS_2$ flakes. To this end, we prepared a ring *without* the $SiO_2$ protective layer. The dimensions of the ring and the FIB parameters are the same as in the rings used for the LP measurements. SEM images of the patterned ring are shown in Fig. S3(a) and (b). We used Electron Backscatter Diffraction (EBSD) to measure the local crystallographic orientation along the circumference of the ring. For the EBSD measurements we used a Zeiss Ultra-Plus FEG-SEM, the electrons energy was set to 10KeV. We estimate the average spatial resolution to be around 100nm. About 90% of the EBSD images were identified to belong to the 194 space group, as expected. 10% of missing or mis-indexed points in an EBSD map is standard even in non-patterned samples.

To show the local orientation we plot in Fig. S3(c-e) the inverse pole figure along the Z, X and Y directions, respectively. The inverse pole figure is a projection of the axes of the sample (where Z is normal to the flake surface, and X and Y are on the surface) on the crystal axes. In (f) we show the color map for the crystal axes. We also measured the orientation on a small square away from the ring area as reference.

We find that the normal to the flake coincides with the (001) direction of the 4Hb-$TaS_2$ crystal as expected. It is clear from the EBSD results that the crystal orientation along the ring is uniform and it is the same orientation found away from the patterned region. Also the amount of missing and mis-indexed pixels is simlar in the ring and in the test area. We can not rule out that there is thin layer of amorphous material along the sample edges [2], but our results show that the ring contains a single crystal ”core” along its entire circumference.

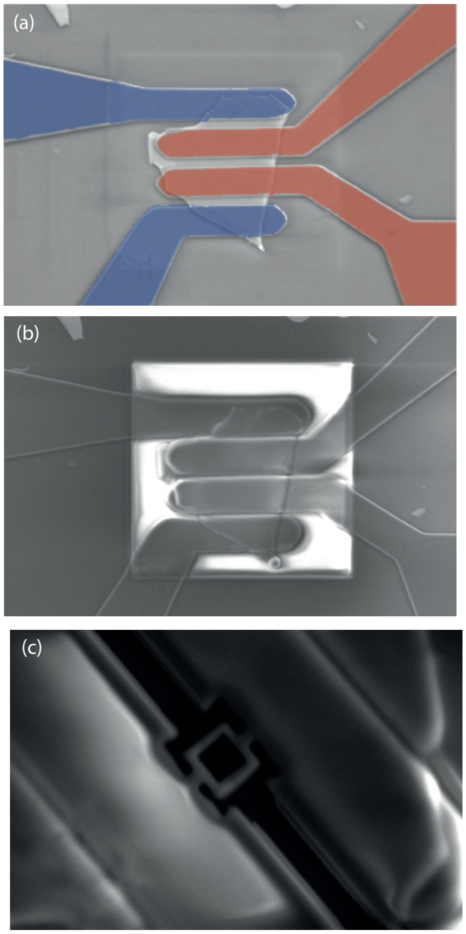


FIG. S1. **Ring Fabrication steps**. SEM images showing the fabrication steps. Shown are images for ring 4 in Fig. S7. (a) Ti/Al contacts evaporated on the 4Hb-$TaS_2$ flake. (b) The white region is the protective $SiO_2$ layer on the flake. (c) The flake is cut using the FIB to create the ring.

## Current dependence

To show that our magneto-resistance measurements were done in the linear regime, we compare the Little-Parks oscillations measured with a current of 300 nA and 800 nA respectively in Fig. S4. As can be seen there is no difference. All the data presented in the paper was measured with a current of less than 300 nA.

## The amplitude of the Little-Parks oscillations

In this section we discuss the amplitude of the resistance oscillations and the estimate of the corresponding amplitude of the $T_c$ oscillations. For the $\pi$-ring presented in Fig.3 of the main text (this corresponds to ring III in the main text, which is ring 6 is Fig. S7), the slope dR/dT is given by 13 m$\Omega$/mK at 2.35K. Taking the area of the ring to be 0.5625 nm$^2$ as measured by SEM, we get a maximum change of 3.5 mK for $T_c$, a period of 36G and an amplitude of 45 m$\Omega$. As in the case of the 0 ring, the calculated period and amplitude are in reasonable agreement with the measured values. In Fig. S5 we show $\Delta R$ for three different temperatures in the temperature range were oscillations are observed, for the same ring.

Using the R(T) of the ring, we can calculate the predicted amplitude of oscillation at 2.37K to be 50 m$\Omega$, at 2.385K to be 60 m$\Omega$ and at 2.39K to be 100 m$\Omega$, compared to the measured amplitudes at these temperatures of 50, 55 and 60 m$\Omega$ respectively.

In addition, using the R(T) curve of sample-I of the main text we can estimate the amplitude of the oscillations at

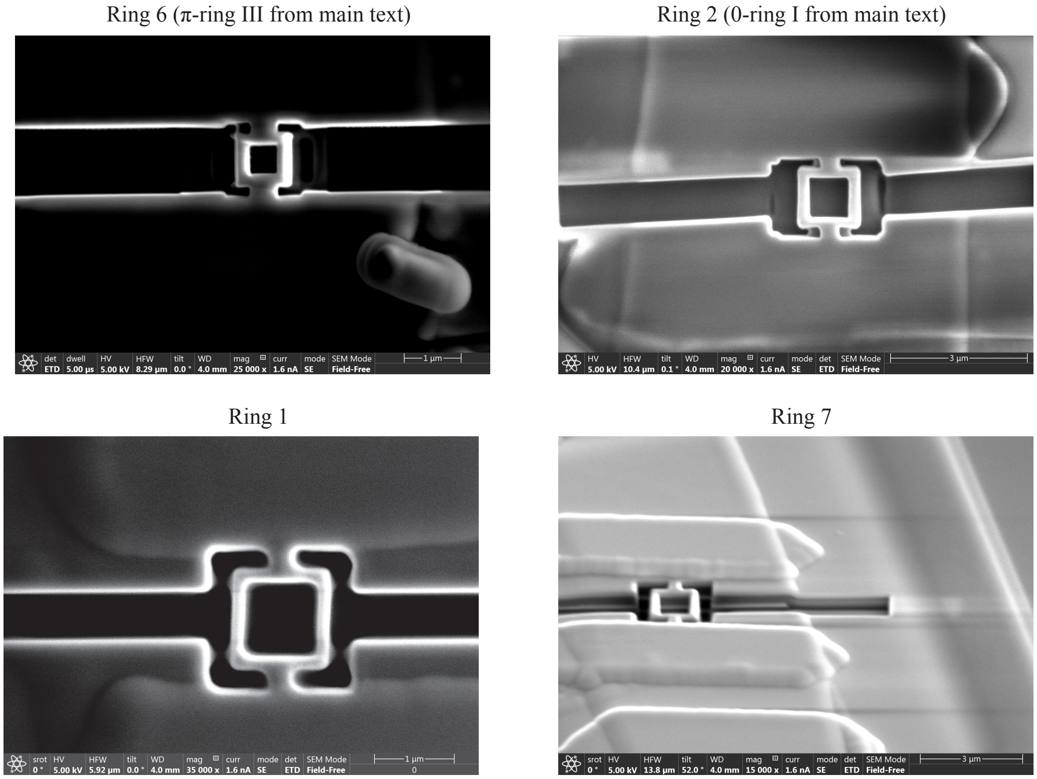


FIG. S2. **SEM images**. Scanning Electron Microscope scan of 4 additional rings. Numbering of the rings is based on Fig. S7
.

different temperatures. Using $\Delta T_c \approx 2.75$ mK with the derivative of R(T) at 2.46, 2.47. 2.48 K (15, 40, 60 m$\Omega$/mK respectively), we find oscillations of $\Delta R \approx$40, 110, 160 m$\Omega$ for these temperatures, in good agreement with the data presented in Fig. 2(d) in the main text.

### Zero-field calibration of the superconducting magnet

A crucial step in our experiment is the accurate determination of the zero-field point of the superconducting magnet, which is used to generate the magnetic flux through the ring. The magnetoresistance, which is symmetric in respect to the field allows us to determine the real zero-field.

For some rings the LP oscillation period is long and the LP background is steep enough to allow an accurate determination of the zero-field.

For other rings we measure the magneto-resistance at a slightly higher temperature where there are no LP oscillations. In Fig. S6 we show the magneto-resistance for 4 rings, in addition to the data shown in Fig. 2c in the main text.

For some rings we measured the residual magnetic field in the system before and after each Little-Parks measurement. In figure S6a and d we show the resistance as function of the field measured before and after the temperature the oscillations were measured. No shift can be observed. This is shown for both a 0-ring (sample 5) and a $\pi$-ring (sample 8). To give an impression of how well the zero is determined we show in Fig. S6c data for a $\pi$-ring (sample III of the main text) the oscillation period of this ring is 36 Oe, clearly the uncertainty in the field is much smaller than half of the oscillation period.

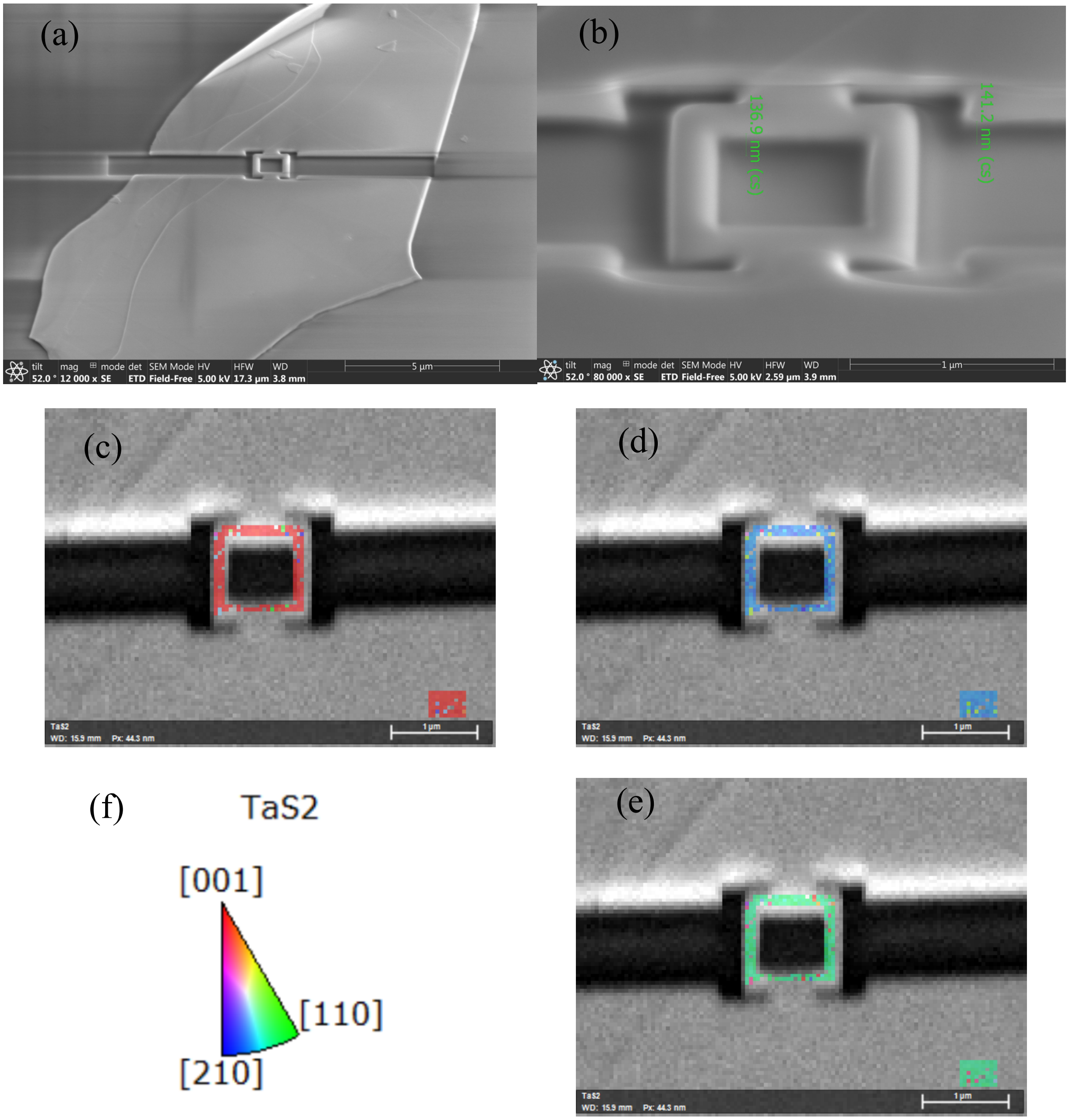


FIG. S3. **EBSD**. (a) and (b) A 4Hb-$TaS_2$ flake not after the FIB carving. For the EBSD test the flake was not covered with the protective SiO layer. (c-e) Inverse pole maps along z, x, and y directions respectively. (f) Color map

## ADDITIONAL DATA

In the following we present additional data that was not presented in the main text.

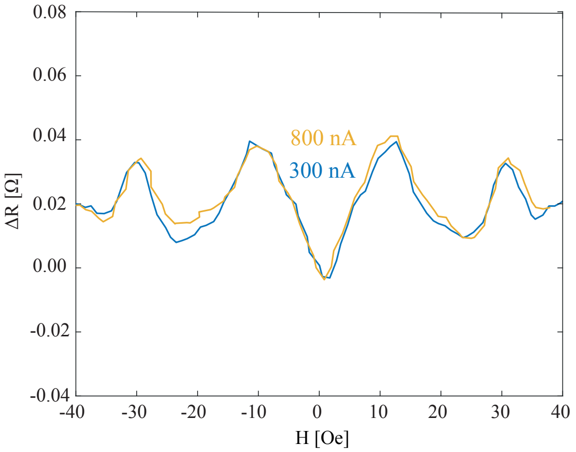


FIG. S4. **Current dependence**. A comparison between the magneto-resistance measured with an AC current of 300nA and 800nA.

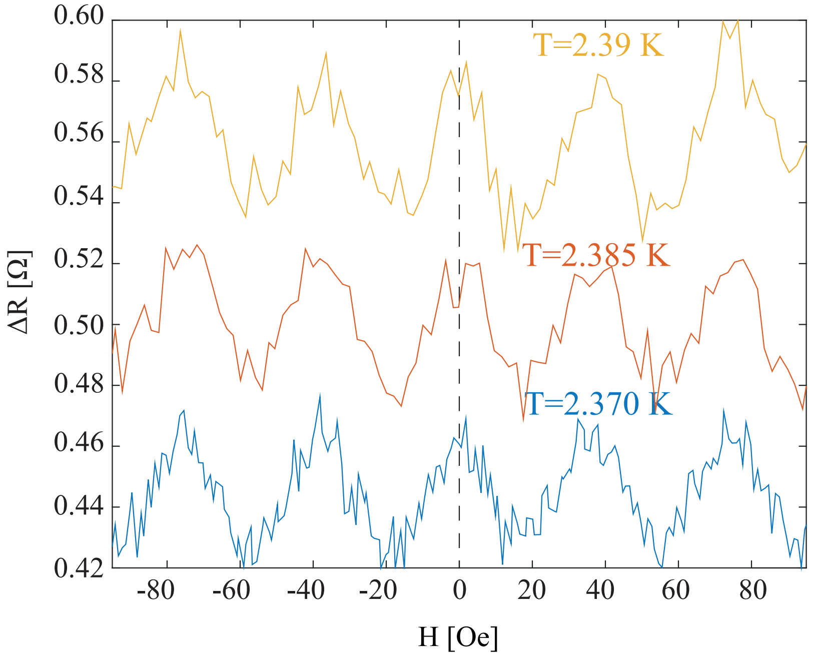


FIG. S5. **Temperature dependence of a $\pi$-ring (Ring III of the main text and ring 6 in Fig. S7)**. LP oscillations at 3 different temperatures.

**Extended data: observation of the Little-Parks oscillations in eight different devices**

We prepared eight rings that showed Little-Parks oscillations. Out of these, three rings are $\pi$-rings. In Fig. S7 we show the data for the eight rings. In the left column we show the resistance as a function of the temperature of the rings, in the middle column we show the magneto resistance and in the right column the Little-Parks oscillations after a background removal.

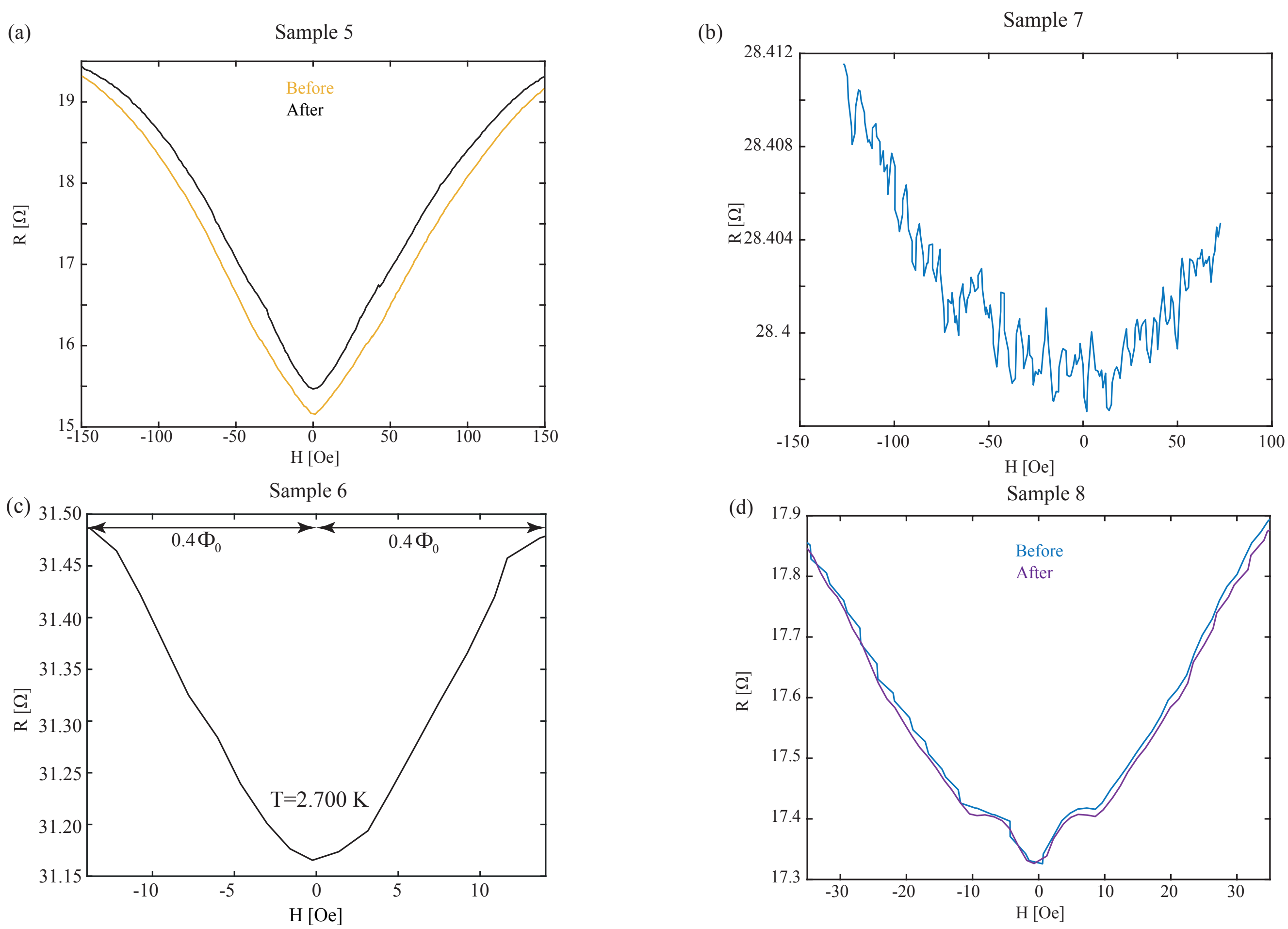


FIG. S6. **Additional background data**. Magneto-resistance data for 4 rings. Data was taken at a temperature above the temperature range at which the LP oscillations were measured. The numbering of the samples is according to Fig. S7. (a) Background measurement before and after LP oscillations measurement, ensuring consistency in the zero magnetic field. (b) Background measurement before the LP oscillations were measured. (c) Background measurement taken before the LP oscillations measurement. Black arrows show the size of 0.4 flux-quanta for this ring. (d) Background measurement before and after LP oscillations measurement. Again showing that the zero magnetic field does not change between in consecutive measurements.

## Thermal cycles

As described in the main text, the behavior of the different rings is reproducible, meaning that a $\pi$-ring will always show a resistance maximum at zero field, even for consecutive cool downs. To demonstrate the stability of the nature of the rings we show in Fig. S8 the magneto-resistance for consecutive cool-downs for a 0 and $\pi$ rings. Between cool downs the rings were heated to 360 K, well above the CDW transition temperature. For the 0-ring this process was repeated 6 times and no change was observed.

## In-plane magnetic field

We measured the Little-Parks oscillations of 3 rings in the presence of an in-plane field. Two out of these rings were 0-rings in the absence of the in-plane field and the third one was a $\pi$-ring. The three samples show the "w" shape that indicates the enhancement of $T_c$ with the application of a small out-of-plane magnetic field. In Fig. S9(a-c) we show the Little-Parks oscillations at three different in-plane field values for a 0-ring( sample II). In Fig. S9d, we show the data for the second 0-ring. The data for the $\pi$-ring is shown in Fig.4 of the main text. It should be noted that the classification of the rings into 0- and $\pi$-rings is based on the existence of the $\pi$-shift *without* an in-plane magnetic

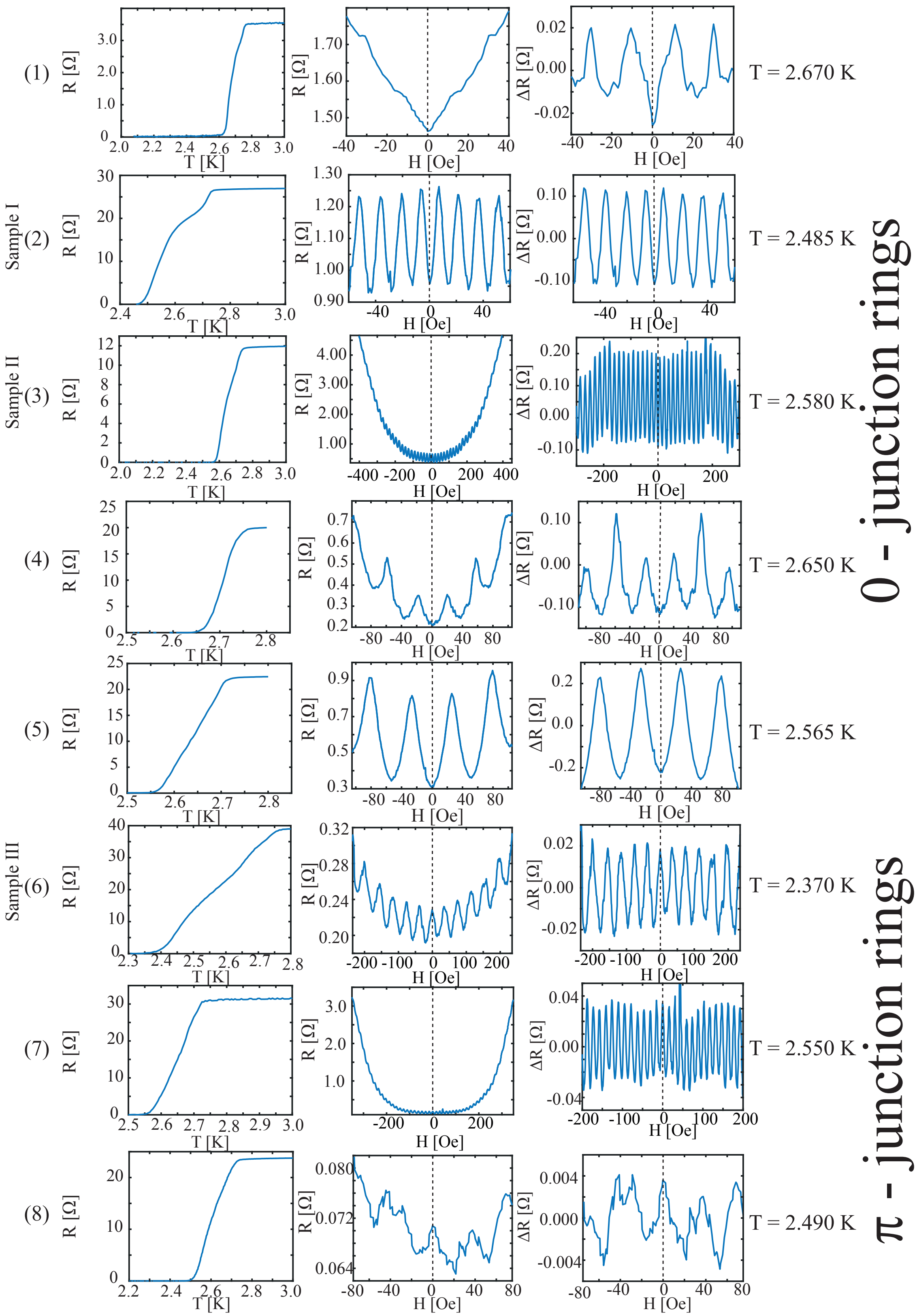


FIG. S7. **Little-Parks oscillations in different rings** We show data for eight rings, five 0-rings and 3 $\pi$-rings. For each ring we show on the left the temperature dependence of the resistance. In the middle row, the magneto-resistance and and the right the Little-Parks oscillations after background subtraction.

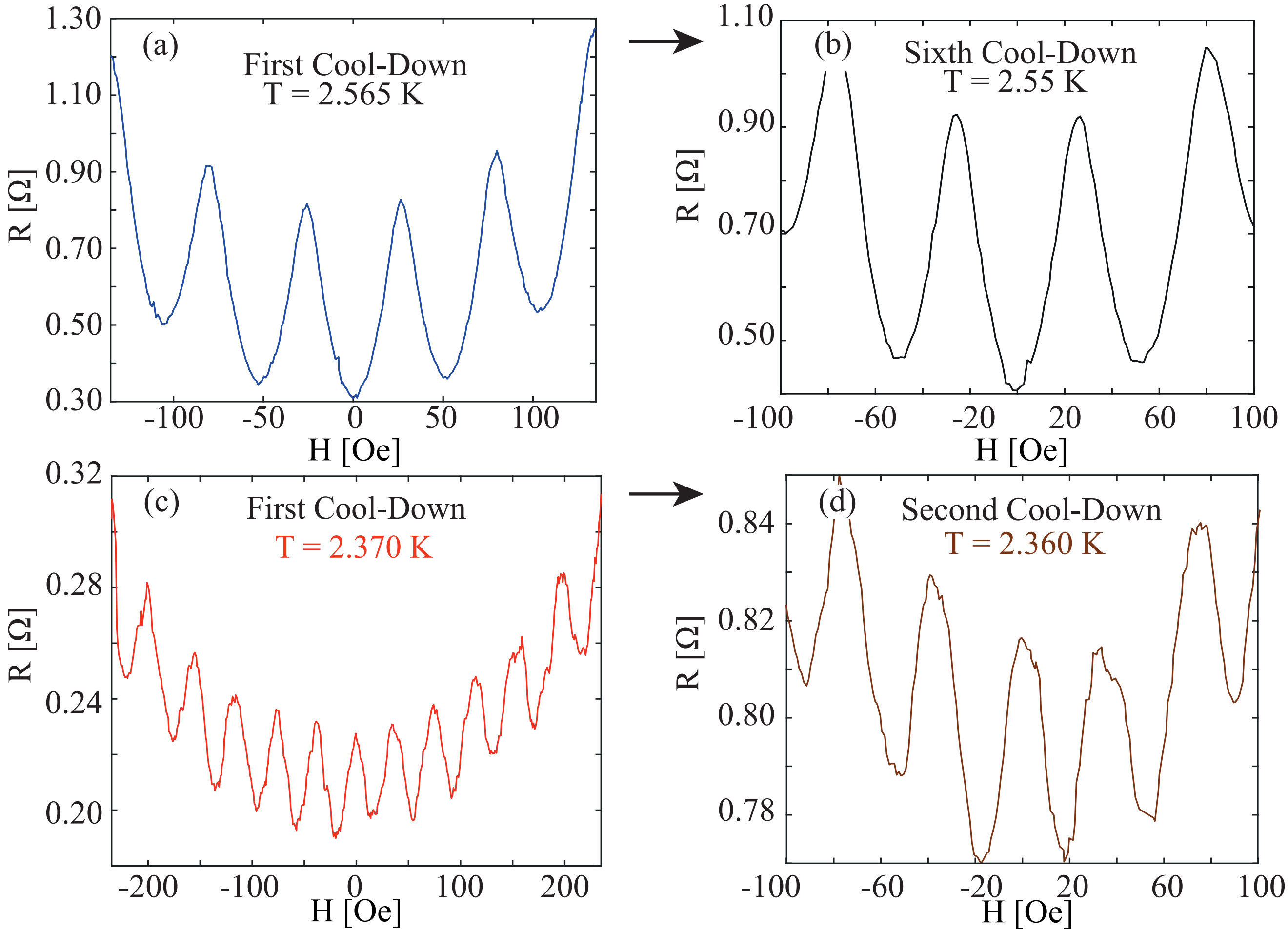


FIG. S8. **Thermal cycles** (a) and (b) magneto-resistance of a 0-ring before and after 6 thermal cycles. (c) and (d) magneto-resistance for a $\pi$-ring before and after a thermal cycle. Between cool-downs the rings were heated to 360K.

field. Applying the in-plane magnetic field can change the value of the "real" out-of-plane zero field because of small misalignment of the in-plane field. For that reason we cannot really tell if a ring changes its character in an in-plane field without re-measuring the magnetoresistance at a higher temperature where LP oscillations are not observed. This was done only for sample II, where indeed no change was found.

## THEORY

In the following, we theoretically discuss the experimental observation of the $\pi$-flux and the enhancement of $T_c$ in a field based on the assumption that the superconducting order parameter belongs to a two-component representation of the symmetry. We also discuss how the observed signatures cannot be explained with a single-component order parameter without fine tuning and/or unrealistic assumptions.

### Ginzburg-Landau Theory of the two-component order parameter

Assuming a two-component order parameter of the form

$$\hat{\Delta}_{\boldsymbol{k}}(\theta,\phi) = \Delta_0 \left[\cos\theta\,\delta_x(\boldsymbol{k}) + e^{i\phi}\sin\theta\,\delta_y(\boldsymbol{k})\right](-i\hat{\sigma}^y)\hat{\sigma}^z\,, \tag{1}$$

which is written in the space of $\Psi^\dagger_{\boldsymbol{k}} = (\psi^\dagger_{\boldsymbol{k}\uparrow}, \psi^\dagger_{\boldsymbol{k}\downarrow})$, such that the order parameter is given by $\langle \Psi^T_{\boldsymbol{k}} \hat{\Delta}(\boldsymbol{k}) \Psi_{-\boldsymbol{k}}\rangle$. Here $\delta_{x,y}(\boldsymbol{k})$ are momentum-dependent basis functions belonging to the two-dimensional irreducible representation $E_{1u}$.

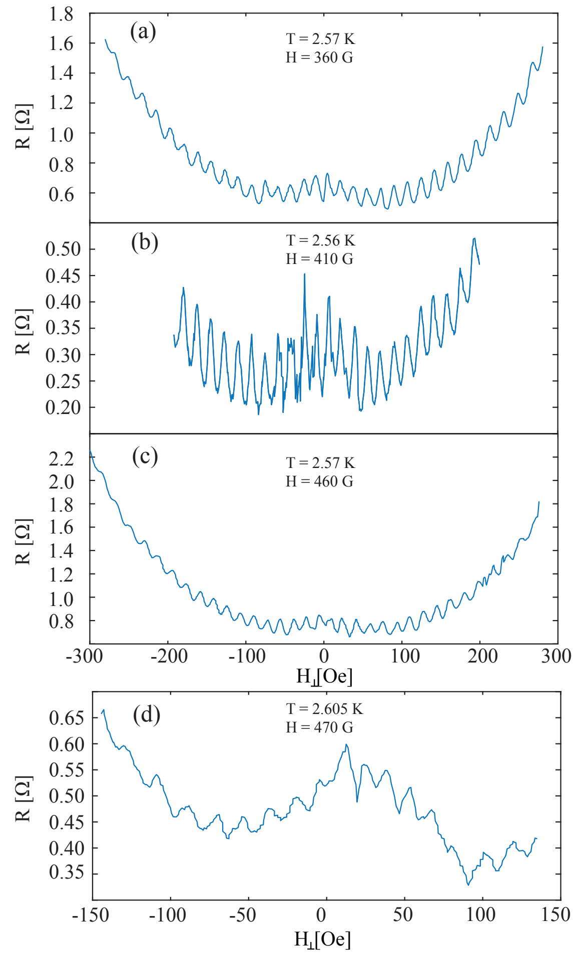


FIG. S9. **LP with in-plane field** (a-c) Little-Parks oscillations with different values of the in-plane magnetic field of a 0-ring. (d) Little-Parks oscillations in the presence of an in-plane field in a second 0-ring.

Note that these transform like the $x$ or $y$ coordinates, but can have a more complicated form. Also, while we have chosen here the spin-triplet (inversion-odd) order parameter for concreteness, the following discussion also holds for an inversion-even order parameter transforming as $E_{2g}$. The angles $\theta$ and $\phi$ are internal degrees of freedom of the order parameter, which will appear in the corresponding Ginzburg-Landau (GL) theory (not to be confused with the superconducting phase, which is implicitly encapsulated in $\Delta_0 = |\Delta_0|e^{i\varphi}$). The correpsonding Ginzburg-Landau free energy density can then be written in terms of a two-component order parameter $\boldsymbol{\eta} = (\eta_1, \eta_2)$, where the two components relate to the above gap function as $\eta_1 = \cos\theta\Delta_0$ and $\eta_2 = e^{i\phi}\sin\theta\Delta_0$. In the following, we thus start from the free energy density

$$f[\boldsymbol{\eta}] = K_1\left(|\boldsymbol{D}\eta_1|^2 + |\boldsymbol{D}\eta_2|^2\right) + K_2|\boldsymbol{D}\cdot\boldsymbol{\eta}|^2 + K_3|\boldsymbol{D}\times\boldsymbol{\eta}|^2 + \alpha(T-T_c)|\boldsymbol{\eta}|^2 + \beta_1|\boldsymbol{\eta}|^4 + \beta_2|\boldsymbol{\eta}^*\times\boldsymbol{\eta}|^2 - \kappa\mathrm{Tr}[\hat{Q}\hat{\varepsilon}]\,, \tag{2}$$

where $\boldsymbol{D} = -i\nabla + e\boldsymbol{A}$ is the (in-plane component of the) covariant derivative (with $c = \hbar = 1$). For simplicity, we have neglected terms that reflect crystal symmetry breaking and assumed a rotationally symmetric model. Finally, the last term, propositional to $\kappa$, describes the coupling to strain, where

$$\hat{Q} = \begin{pmatrix} |\eta_1|^2 - |\eta_2|^2 & \eta_1^*\eta_2 + c.c. \\ \eta_1^*\eta_2 + c.c. & |\eta_2|^2 - |\eta_1|^2 \end{pmatrix}$$

and

$$\hat{\varepsilon} = \begin{pmatrix} \varepsilon_{xx} - \varepsilon_{yy} & \varepsilon_{xy} \\ \varepsilon_{xy} & \varepsilon_{yy} - \varepsilon_{xx} \end{pmatrix}.$$

Previous experiments [3–5] are consistent with a fully gapped and chiral superconducting state. We may assume that $\beta_2 < 0$. Moreover, we note that for any $K_2 \neq -K_3$ there is a linear coupling between the transverse magnetic field and the superconducting order parameter.

### The Little-Parks effect in uniform strain

We first analyze the Little-Parks effect in the presence of uniform strain along $\hat{\boldsymbol{x}}$, namely $\varepsilon_{xx} = -\varepsilon_{yy} = \varepsilon_0/2$ and $\varepsilon_{xy} = 0$. Moreover, we assume the ring to be annular with inner and outer radii given by $R_1$ and $R_2$, respectively. Finally, we first neglect coupling of order parameters by the magnetic field, which is achieved by setting $K_2 = -K_3$. We will relax this assumption in the next subsection.

Close to $T_{\rm c}$, where the quartic terms are negligible, and in the absence of a magnetic field, the order parameter will be aligned by the strain term, such that we have $\eta_1 = \eta_0$ and $\eta_2 = 0$. The resulting free energy density then takes the form

$$f[\boldsymbol{\eta}] = (K_1 + K_2)|D_x\eta_1|^2 + (K_1 - K_2)|D_y\eta_1|^2 + \left[\alpha(T - \tilde{T}_c) + \kappa\varepsilon_0\right]|\eta_1|^2 + \mathcal{O}(\eta^4)\,. \tag{3}$$

We can include a magnetic field via the gauge choice $\boldsymbol{A} = H(-y\hat{\boldsymbol{x}} + x\hat{\boldsymbol{y}})/2 = Hr/2\hat{\boldsymbol{\gamma}}$ in cylindrical coordinates with $\gamma$ the azimuth. We also assume that the gap function does not depend on the radius and has a winding number $n$, such that $\eta_0 = |\eta_0|e^{in\gamma}$. The free energy up to quadratic order is then given by

$$F = \int r dr d\gamma dz f[\eta_0] = \alpha\pi(R_2^2 - R_1^2)h\left[T - \tilde{T}_{\rm c}(H)\right]|\eta_0|^2, \tag{4}$$

where $h$ is the height of the ring and

$$\tilde{T}_{\rm c}(H) = T_{\rm c} + \frac{\kappa\varepsilon_{xx}}{\alpha} - g\int_{R_1}^{R_2}\frac{dr}{r}\left(n - \frac{eHr^2}{2}\right)^2, \tag{5}$$

with $g = K_1/\alpha(R_2^2 - R_1^2)$. As usual, $n$ is chosen to minimize $F$ (or maximize $\tilde{T}_{\rm c}$).

Figure S10 shows Eq. (5) with flux $\Phi/\phi_0$, where $\Phi \equiv \pi R_1^2 H$. We use the radii ratio $R_1/R_2 = 0.85$ and set the parameters $g = 0.01(T_{\rm c} + \kappa\varepsilon_{xx}/\alpha)$. The parameters are chosen to fit the dimensions of the ring in the experiment and the size of the oscillations (order $10^{-3}T_{\rm c}$). Interestingly, the strength of the parabolic envelope and the size of the oscillations are not independent of each other. As can be seen in the figure, the parabolic envelope of the oscillations fits reasonably well to experiment (see main text). Namely, $\Delta T_{\rm c}/T_{\rm c}$ reaches about $-10^{-3}$ after an order of 10 oscillations. This shows that the main origin of the parabolic magnetoresistance comes indeed from the finite width of the ring.

As a final note, we mention that Eq. (4) with $K_2 = 0$ describes any single-component order parameter, in other words an order parameter transforming like a one-dimensional irreducible representation. This is not surprising, as so far we have described a 0-flux ring.

### The Little-Parks effect in a chiral state

Another simple limit, is the case where there is no strain. Then, the magnetic field will naturally select the chiral state (assuming that $K_2 \neq -K_3$). In this case, $\eta_1 = \eta_0$ and $\eta_2 = \pm i\eta_0$, where $\pm$ denotes the chirality. The GL free energy density now assumes the form

$$f[\eta_0] = (2K_1 + K_2 + K_3)|\boldsymbol{D}\eta_0|^2 - e|H|(K_2 + K_3)|\eta_0|^2 + \alpha(T - T_{\rm c})|\eta_0|^2 + \mathcal{O}(\eta^4)\,, \tag{6}$$

where the absolute value of $H$ comes from choosing the chiral state, which is favored by the magnetic field.

As before, we will assume that $\eta_0 = |\eta_0|e^{in\gamma}$ and $\gamma$ is the azimuth. Integrating over the volume of the ring, we obtain Eq. (4) with $\tilde{T}_c(H)$ given by

$$\tilde{T}_{\rm c}(H) = T_{\rm c} + \frac{2\eta_0^2(K_2 + K_3)}{\alpha R_1^2}\frac{|\Phi|}{\phi_0} - g'\int_{R_1}^{R_2}\frac{dr}{r}\left(n - \frac{eHr^2}{2}\right)^2, \tag{7}$$

where $g' = (2K_1 + K_2 + K_3)/2\alpha(R_2^2 - R_1^2)$.

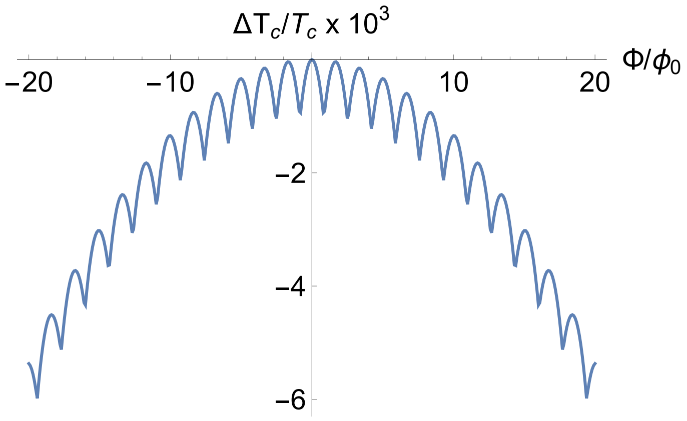


FIG. S10. The value of $\tilde{T}_c$ (5) vs. magnetic flux $\Phi/\phi_0$. Here we used $g = 0.01(T_c + \kappa\varepsilon_{xx}/\alpha)$ and $R_1/R_2 = 0.85$. Also note that $\Phi \equiv \pi R_1^2 H$. Notice that $\Delta T_c/T_c$ reaches $\sim -5 \times 10^{-3}$ after 10 oscillations, in good agreement with experiment.

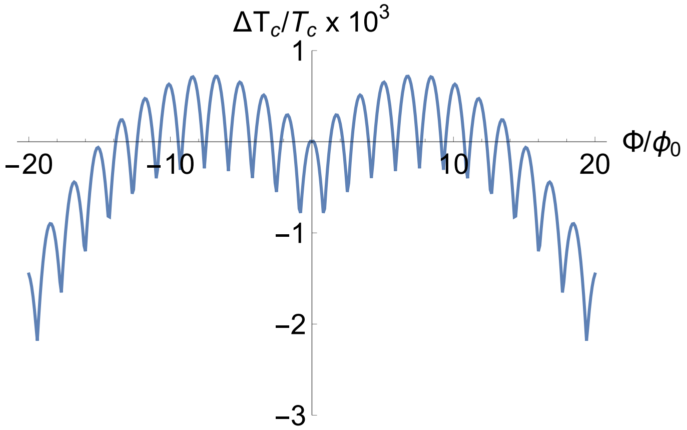


FIG. S11. Eq. (18) vs. $|\Phi|/\phi_0$ for $g' = 0.01T_c$ and $K_2 + K_3 = 2 \times 10^{-4}\alpha R_1^2 T_c$. This result should be compared with panel (b) of Fig.4.

In Fig. S11 and S12 we plot Eq. (18) for $g' = 0.01T_c$, $K_2 + K_3 = 2 \times 10^{-4}\alpha R_1^2 T_c$ and $K_2 + K_3 = 0.5 \times 10^{-4}\alpha R_1^2 T_c$, respectively. Fig. S11 shows an enhancement in $T_c$, seen when a magnetic field is applied. This resembles the situation in Fig. 4, where an in-plane magnetic field is applied. Fig. S12 shows the same effect with a weaker value of $K_2 + K_3$. This plot should be compared with panel (a) of Fig. 2. Indeed, we find that the parabolic dependence near zero field in Fig. 2 is flatter than the expected behavior for a non-chiral order parameter Fig. S10.

As such, while the difference between the values of the parameter $K_2+K_3$ in the two figures explains the experiment, it is highly unlikely that an in-plane magnetic field causes such a shift. The parameters of the GL free energy are typically set by the non-interacting band structure which is not affected by such a small field. It is much more likely that the in-plane magnetic affects the competition between the strain, which prefers a real order parameter, and the out-of-plane magnetic field, which prefers a chiral state.

### The chiral-nematic mixed state in the presence of strain and magnetic field

In the previous two subsections, we have chosen gap functions that are either purely real with $\phi = 0, \pi$ ("nematic") or $\phi = \pi/2$ ("chiral"). We now comment on the more realistic situation of both non-zero strain and $K_2 \neq -K_3$. Without an out-of-plane magnetic field, the degeneracy of the order-parameter is broken and the order parameter will be 'aligned' with the strain and TRS is preserved as discussed above. However, the coupling to a TRS-breaking order parameter through an out-of-plane magnetic field is still present in the free energy, and as such the system can still have an increased $T_c$ at small fields. Specifically, $T_c$ is controlled by the quadratic GL free energy density (neglecting

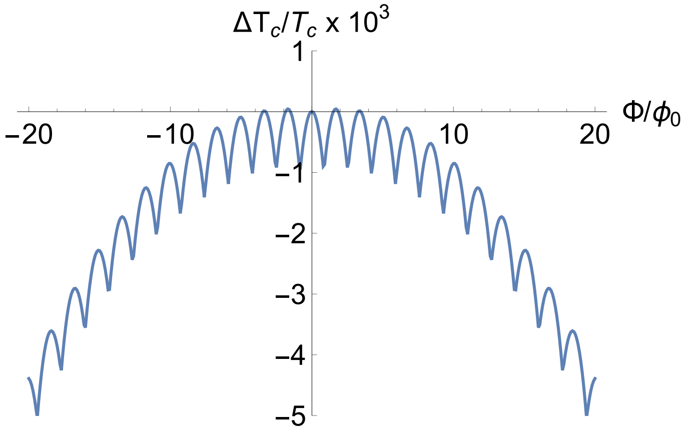


FIG. S12. Eq. (18) vs. $|\Phi|/\phi_0$ for $g' = 0.01T_c$ and $K_2 + K_3 = 0.5 \times 10^{-4}\alpha R_1^2 T_c$

spatial variations of the gap)

$$f = \boldsymbol{\eta}^\dagger \begin{pmatrix} \alpha(T - T_c) - \kappa\varepsilon_{xx} & ie(K_2 + K_3)H \\ -ie(K_2 + K_3)H & \alpha(T - T_c) + \kappa\varepsilon_{xx} \end{pmatrix} \boldsymbol{\eta} + \mathcal{O}(\eta^4)\,, \tag{8}$$

where we have assumed the strain is uniform and along the $x$ direction.

$T_c$ is given by the largest negative eigenvalue of the matrix, that is

$$\tilde{T}_c = T_c + \frac{1}{\alpha}\sqrt{\kappa^2\varepsilon_{xx}^2 + e^2(K_2 + K_3)^2 H^2}. \tag{9}$$

Thus, due to the competition with strain, the dependence of $T_c$ on magnetic field will be quadratic close to $H = 0$, as apposed to the linear dependence assumed in Eq. (18). The state right below $T_c$ is then a "nematic-chiral" mixture with $\phi = \pm\pi/2$ (depending on the direction of the strain field) and

$$\cos\theta = \frac{1}{\sqrt{2}}\left[1 + \frac{\kappa\varepsilon_{xx}}{\sqrt{\kappa^2\varepsilon_{xx}^2 + e^2(K_1 + K_2)^2 H^2}}\right]^{1/2}.$$

This mixed state is expected to have a weaker positive contribution to $T_c$ due to the chiral state as compared to Eq. (18). Namely, when $eH(K_2 + K_3) \ll \kappa\varepsilon_{xx}$ this contribution will be quadratic in $H$. Given that $T_c$ is expected to increase as $H^2$ due to the finite width of the ring, this chiral contribution is only expected to reduce the coefficient in front of $H^2$, which will still be positive, thus not showing an overall $T_c$ enhancement.

Again, we do not know the origin of the $T_c$ enhancement when an in-plane field is present. However, we can speculate that if the in plane field suppresses the coupling to strain then this quadratic contribution can be converted to a dominant linear contribution as in Fig. 18 when the in-plane field is present. On the other hand, when it is not present, the strain is dominant thus suppressing the $T_c$ enhancement.

Finally, note that a coupling of the magnetic field to two order parameters is more generally allowed. In particular, noting that the field transforms as $A_{2g}$, any order parameter combination that transforms as $A_{2g}$ is allowed, since $A_{2g} \otimes A_{2g} = A_{1g}$, in other words the full combination transforms as a scaler. As an example, an order parameter of $B_{1u}$ symmetry can couple to one with $B_{2u}$ symmetry through the magnetic field. For combinations of higher-dimensional irreps, the decomposition needs to contain $A_{2g}$. This is in particular possible for the two-dimensional irreps relevant for 4Hb-$TaS_2$ , since $E_{2g} \otimes E_{2g} = A_{1g} \oplus A_{2g} \oplus E_{2g}$ and $E_{1u} \otimes E_{1u} = A_{1g} \oplus A_{2g} \oplus E_{1u}$. As just noted, the effect will only be appreciable, if the two coupled order parameters are very close in energy, in other words have (almost) degenerate $T_c$. While this is naturally given for the two-dimensional irreps even with a small perturbation such as strain, an $f$-wave order parameter has no natural partner in this system, and no increase in $T_c$ can be expected.

### Possible scenario for half-vortices of the planar strain field: The strain induced by a dislocation

In this section we seek the fundamental solution of stress in a two-dimensional ring geometry in the presence of a dislocation defect at its center. This solution can be derived from the stress field of a disclination, which obeys the

equation

$$(\nabla^2)^2\chi = q\delta(\boldsymbol{r})\,, \tag{10}$$

where the stress is related to the scalar field $\chi$ via a second derivative of the form

$$\sigma^{ik} = \epsilon^{ij}\epsilon^{kl}\partial_j\partial_l\chi\,. \tag{11}$$

Then strain is related to stress in the standard manner

$$\varepsilon_{ij} = A_{ijkl}\sigma^{kl}. \tag{12}$$

From equation Eq. (10) we obtain the fundamental solution of a dislocation by noting that a dislocation is a dipole of disclinations. Assuming the dislocation is a dipole along the $x$-axis we obtain such an expression using

$$\chi' = \frac{b}{q}\partial_x\chi\,.$$

The solution of Eq. (10) is given by

$$\chi(r,\gamma) = c_1\log r + c_2 r^2 + c_3 + \frac{qr^2}{8\pi}(\log r - 1/2) \tag{13}$$

Taking the derivative with respect to $x$ we then obtain

$$\chi'(r,\gamma) = \left[\frac{\tilde{c}_1}{r} + \tilde{c}_2 r + \frac{br}{4\pi}\log r\right]\cos\gamma \tag{14}$$

Using Eq. (11) we can then obtain the stress tensor. Finally, the values of the constants $\tilde{c}_1, \tilde{c}_2$ are dictated by the boundary conditions that require a vanishing stress normal to the boundary $\sum_j \sigma^{ij} n_j = 0$.

The resulting strain field is plotted in Fig. S14. Here we used a "fat" ring to make the strain texture visible. The topology in a thin ring remains the same. As can be seen, the texture is more complex than the one depicted in the main text. The boundary conditions induce two additional topological defects (red dots). It is evident that their topological charge is also halved. Indeed, any contour going around the hole and only one of the defects has an even number of branch cuts.

A suggested covering of this strain field with a two-component (real) order parameter is presented. The red lines mark branch cuts across which the order parameter field can not be glued and must change sign. As a consequence it will vanish near this region and develop a spontaneous $\pi$ junction. When a $\pi$ flux is introduced to the center hole this frustration is removed, thus causing $T_c$ to increase. This will manifest itself as a $\pi$ shift in the Little-Parks oscillations.

**Topological classification of the strain field around the ring and its relevance to the Little-Parks experiment**

The fact that $\pi$-junctions appear in certain samples regardless of temperature cycles above the CDW transition is a key experimental observation. It implies the origin of $\pi$ junction is very likely a structural one. A second key observation is that the $\pi$ shift is quite common (observed in roughly half of the devices).

In the main text, we have argued that strain fields in the sample can align the two-component order parameter close to $T_\mathrm{c}$ via the $\kappa$-term in the GL free energy density Eq. (2). In this way, the strain field orientation, which is fixed by the ring geometry and structure, is embedded into the order parameter. Strain may frustrate the order parameter and force it to develop a $\pi$-shift [1], similar to the substrates in Ref. [6], which fixed the different crystallographic axis. Indeed, a two-component order parameter has been shown to be very sensitive to strain [7, 8]. Moreover, strain is expected to be a sample dependent feature explaining the stability of the $\pi$-flux to thermal cycles.

The question that remains is thus, what kind of strain field is required to force a $\pi$ shift and what can lead to such a field in half of our samples? The simplest scenario is depicted in Fig. 3. (For completeness we also schematically present the $\pi$-junction formation for a $E_{2g}$ order parameter in Fig. S13.) The strain, which in 2D is described by a headless vector (an axis), can rotate by $\pi$ around the ring, without causing any inconsistency (like a disclination in a nematic medium). Unlike strain however, the order parameter is not compatible with such a rotation and is thus frustrated by such a configuration.

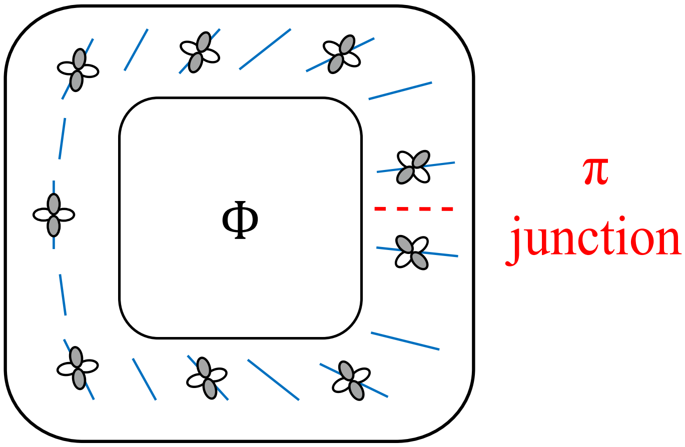


FIG. S13. The formation of a $\pi$ junction for a "d-wave" ($E_{2g}$) order parameter. Note that the coupling to strain has the same form as for the $E_{1u}$ order parameter. In this case, the order parameter only "rotates by $\pi/4$" when the strain rotates by $\pi/2$.

To show this we assume a simple strain field coupling of the form

$$\delta f \sim -\kappa[(|\eta_1|^2 - |\eta_2|^2)\cos\gamma + (\eta_1^*\eta_2 + \eta_2^*\eta_1)\sin\gamma]\,, \tag{15}$$

where $\gamma$ is the azimuth. Close to $T_c$, this term will locally affect $T_c$. The order-parameter configuration with highest transition temperature is then

$$\boldsymbol{\eta} = \eta_0(\delta_x\hat{\boldsymbol{x}} + \delta_y\hat{\boldsymbol{y}})\,, \tag{16}$$

where $\delta_x = \cos\gamma/2$ and $\delta_y = \sin\gamma/2$. Also, we define $\eta_0 = e^{i\varphi}|\eta_0|$, where $\varphi = \gamma(n+1/2)$ and $n \in \mathbb{Z}$ to ensure the order parameter is single-valued. We note that this configuration has the highest $T_c$ conditioned that the ring circumference is large enough compared to the coherence length. Namely, we need to assume that $K_1, K_2, K_3 \ll R_1\kappa\varepsilon$, which is achieved in the large ring limit $R_1 \gg \xi$.

Plugging this ansatz in quadratic gradient terms of Eq. (2) we obtain

$$f_2 \sim \left[K_1 + K_2(\boldsymbol{\delta}\times\hat{\boldsymbol{r}})^2 + K_3(\boldsymbol{\delta}\cdot\hat{\boldsymbol{r}})^2\right]|(-i\nabla + e\boldsymbol{A})\eta_0|^2 + \frac{|\eta_0|^2}{r^2}\left[K_1 + K_2(\partial_\gamma\boldsymbol{\delta}\times\hat{\boldsymbol{r}})^2 + K_3(\partial_\gamma\boldsymbol{\delta}\cdot\hat{\boldsymbol{r}})^2\right] \tag{17}$$

Assuming for simplicity that $K_2 = K_3$ we obtain

$$\tilde{T}_c(H) = T_c + \delta T_c - g''\int_{R_1}^{R_2}\frac{dr}{r}\left(n + 1/2 - \frac{eHr^2}{2}\right)^2\,, \tag{18}$$

where $\delta T_c$ is a flux independent shift coming from the second term on the right hand side of Eq. (17) and the coupling to strain Eq. (16) and $g'' = (K_1 + K_2/2 + K_3/2)/2\alpha(R_2^2 - R_1^2)$. This expression manifestly exhibits a $\pi$ phase shift compared to the oscillations shown in Fig. S10.

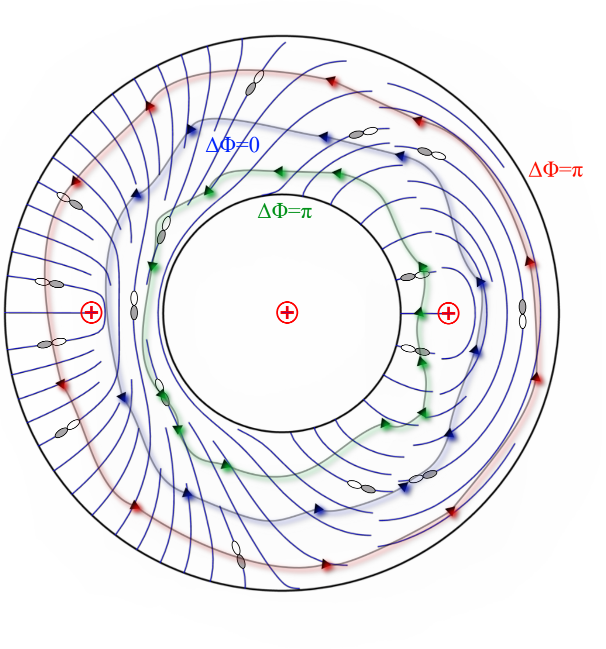


FIG. S14. Strain field obtained from Eq. (14) of a two-dimensional ring with inner and outer radii ratio $R_1/R_2 = 0.42$. Here a dislocation of arbitrary strength is assumed to be placed at the origin. Its presence generates a half-integer topological defect in the strain field structure, which schematically denoted by the red "+" charge. The boundary conditions force the strain field to develop two additional half-integer defects located along the $y = 0$ (schematically represented by the red "+" charges). Three possible semi-classical paths for a Cooper pair encircling the hole are denoted. Those paths that go around one or three defects entail a $\pi$-shift on the order parameter, while any path that goes around two defects (only one of the negative charges) will not induce a $\pi$-shift. The picture emerging from this structure is thus more complex and requires further analysis, which is beyond the scope of the current paper.

* amitk@physics.technion.ac.il

[1] Geshkenbein, V. B., Larkin, A. I. & Barone, A. Vortices with half magnetic flux quanta in "heavy-fermion"superconductors. *Physical Review B* **36**, 235 (1987).
[2] Moll, P. J. Focused ion beam microstructuring of quantum matter. *Annual Review of Condensed Matter Physics* **9**, 147–162 (2018).
[3] Ribak, A. *et al.* Gapless excitations in the ground state of 1 t- tas 2. *Physical Review B* **96**, 195131 (2017).
[4] Nayak, A. K. *et al.* Evidence of topological boundary modes with topological nodal-point superconductivity. *Nature physics* **17**, 1413–1419 (2021).
[5] Persky, E. *et al.* Magnetic memory and spontaneous vortices in a van der waals superconductor. *Nature* **607**, 692–696 (2022).
[6] Tsuei, C. *et al.* Pairing symmetry and flux quantization in a tricrystal superconducting ring of $YBa_2Cu_3O_{7-\delta}$. *Physical Review Letters* **73**, 593 (1994).
[7] Hicks, C. W. *et al.* Strong increase of $t_c$ of $Sr_2RuO_4$ under both tensile and compressive strain. *Science* **344**, 283–285 (2014).
[8] Yuan, A. C., Berg, E. & Kivelson, S. A. Strain-induced time reversal breaking and half quantum vortices near a putative superconducting tetracritical point in $Sr_2RuO_4$. *Physical Review B* **104**, 054518 (2021).
